# Association of MDR1 C1236T Polymorphisms and B Cell Non-Hodgkin Lymphoma

**DOI:** 10.7150/jca.66312

**Published:** 2022-03-14

**Authors:** Wei Zhang, Qun Zhang, Hui Liang, SuLi Wang, Jing Pan, ShaoYing Pan, Bin Zhu, ZhiYong Ding, WenLi Zhao, Ying Ni

**Affiliations:** 1 Department of Radiotherapy, Fengcheng Hospital of Fengxian District, Shanghai 201411, China.; 2 Department of Oncological Surgery, Minhang Branch of Fudan University Shanghai Cancer Center, Shanghai 200240, China.; 3 Department of General Surgery, Naval Medical Center of PLA, Shanghai 200052, China.; 4 Department of Hematology, Shanghai Jiao Tong University Affiliated Sixth People's Hospital South Campus, Shanghai Fengxian District Central Hospital, Shanghai 201499, China.; 5 Department of Hematology, Anhui University of Science and Technology Affiliated Fengxian Hospital, Shanghai 201499, China.

**Keywords:** B-cell non-Hodgkin lymphoma, multidrug resistance 1 (MDR1), polymorphism, treatment-related toxicities

## Abstract

**Background:** Multidrug resistance gene 1 (MDR-1) encodes for P-glycoprotein (P-gp) recognized for removing cytostatic drugs from tumor cells. MDR1 gene polymorphisms change function of P-gp. In this study, we are interested in investigating whether MDR1 C1236T single nucleotide polymorphisms (SNPs) affect the susceptibility and treatment-related toxicities in B-cell non-Hodgkin lymphoma (B-NHL) in the population of eastern China.

**Materials and methods:** A group of 107 B-NHL patients and 150 healthy donors, unrelated ethnic Han Chinese and residents of eastern China, were included in this study. The MDR1 C1236T polymorphisms were determined using polymerase chain reaction-allele specific primers after extraction of genomic DNA. Analyses were performed using SPSS and Arlequin software.

**Results:** MDR1 C1236T polymorphisms were not significantly related to the risk and treatment-related toxicities of B-NHL. A significant association between extranodal sites and C1236T allele was observed (C vs T: P=0.01).

**Conclusion:** Our findings could expand our understanding of MDR1 in B-NHL and provide references for further research in multidrug resistance.

## Introduction

B-cell non-Hodgkin lymphoma (B-NHL) comprises a heterogeneous group of cancers and accounts for 85-90% of non-Hodgkin's lymphoma (NHL) 1,2. It was estimated that there were 81,560 new NHL cases and 20,720 deaths in the United States in 2021 3. These malignancies usually develop in the lymph nodes, but can involve any tissue in the body 1. Patients present with painless lymphadenopathy, and a diverse of clinical symptoms dependent on the site of involvement 1,4. Diagnosis of B-NHL is based on pathology of an excisional biopsy. The Ann Arbor classification is used to determine the stage of lymphoma 5. The International Prognostic Index (IPI) and the age-adjusted International Prognostic index is used in predicting long-term survival for patients 6. Main treatments for B-NHL are chemotherapy, immunochemotherapy, and radiation therapy 7-9. However, most of these malignancies are refractory or relapsed after initial treatment.

Multidrug resistance gene 1 (MDR-1), also known as the adenosine triphosphate binding cassette B1 gene (ABCB1), is located on chromosomal region 7q21.1 10. MDR1 encodes for a transmembrane protein of 170 kD named P-glycoprotein (P-gp), recognized and named for removing cytostatic drugs from tumor cells 10-13. The function of P-gp is changed by MDR1 gene polymorphisms. Patients with these variants are at an increased risk of developing a variety of diseases and have different response to same treatment regimens 12. To date, more than 50 single nucleotide polymorphisms (SNPs) of MDR1 have been described 14. Among these reported SNPs, few studies focus on association between C1236T polymorphism and treatment-related toxicities in B-NHL.

In this study, we are interested in investigating whether MDR1 C1236T SNPs affect the susceptibility and treatment-related toxicities in B-NHL in the population of eastern China. Our findings could expand our understanding of MDR1 in B-NHL and provide references for further research in multidrug resistance.

## Materials and methods

### Subjects

A group of 107 B-NHL patients and 150 healthy donors were included in this study. All Subjects were unrelated ethnic Han Chinese, residents of Shanghai, Jiangsu and the surrounding regions. Patients were dignosed based on pathology according to 2008 and 2016 World Health Organization (WHO) classification for tumors of hematopoietic and lymphoid tissues 15,16 from April 2014 to May 2020. Patients were excluded if they had concurrent cancer, severe cardiohepatic and renal dysfunction, severe cardiovascular and cerebrovascular disease, or they were during pregnancy or lactation. They were treated with R-CHOP or R-CHOP-like regimens as initial induction chemotherapy. Complete blood cell counts and toxicity assessments were performed after each cycle of chemotheraphy. Hematologic toxicities (leukopenia, neutropenia, anemia and thrombocytopenia) were measured using the Common Terminology Criteria for Adverse Events version 3.0. The study adhered to the principles of the Helsinki Declaration.

### Genotyping

Genomic DNA was extracted manually from peripheral blood. The MDR1 C1236T polymorphism was determined using the polymerase chain reaction-allele specific primers assay. Each polymerase chain reaction (PCR) reaction mixture contained: a PCR Master Mix reaction buffer (10 μl), DNA template (1 μl), primer (2 μl) and double distilled water (7 μl). For allele T, forward primer is 5-GTCACTTCAGTTACCCATCTCG-3 and reverse primer is 5-CTGCACCTTCAGGTTCGGA-3. For allele C, forward primer is 5-GTCACTTCAGTTACCCATCTCG-3 and reverse primer is 5- CTGCACCTTCAGGTTCTGG-3. For T/C, forward primer is 5-GTCACTTCAGTTACCCATCTCG-3 and reverse primer is 5- GGTCATAGAGCCTCTGCATCA-3. The PCR conditions were 10 cycles of denaturation at 96 °C for 10 min, annealing at 58 °C for 30 min, and extension at 60 °C for 75 min, and 25 cycles of denaturation at 96 °C for 10 min, annealing at 55 °C for 30 min, and extension at 60 °C for 75 min. DNA fragments generated after restriction enzyme digestion were separated in a 2% agarose gel. The C1236T polymorphism of MDR1 was genotyped homozygous CC genotype, homozygous TT genotype and heterozygous CT genotype.

### Statistical analysis

The Hardy-Weinberg equilibrium test was performed by Arlequin software 3.5. Associations between allele, genotype frequencies of MDR1 polymorphism and risk of B-NHL patients were determined using Binary logistic regression analysis. Pearson Chi-squared and Fisher's exact test were used to analyze association between patients' MDR1 polymorphism and clinical characteristics, treatment-related toxicities. All tests were two-sided and differences were considered significantly when p-value was <0.05. All analyses were performed using SPSS for Windows (version 26.0, SPSS, Chicago, IL, USA).

## Results

### Patient characteristics

The study consisted of 107 B-NHL patients. 56 were men and 51 were women. 52.3% patients were ≤60 years old. Clinical characteristics, including sex, age, staging, grouping, extranodal sites, ECOG score, LDH and IPI score, are shown in Table 1.

### Hardy-Weinberg equilibrium examination

Table 2 shows examination of Hardy-Weinberg equilibrium of MDR1 SNPs (C1236T at rs1128503) in both B cell lymphoma groups and control groups. P value was > 0.05. The SNPs of two groups were both in accordance with Hardy-Weinberg equilibrium.

### Association between MDR1 polymorphisms and risk of B-NHL

To estimate the association between MDR1 polymorphisms and the risk of developing B-NHL, Binary logistic regression analysis was used. For MDR1 1236T allele, 70.1% were in patients and 64.7% in controls (OR=1.28, 95% CI=0.88-1.87, p= 0.2). For MDR1 1236CT genotype, 46.7% were in patients and 45.3% in controls (OR=2.00, 95% CI=0.78-5.11, p= 0.15), and for MDR1 1236TT allele, 46.7% were in patients and 42% in controls (OR=2.15, 95% CI=0.84-5.53, p= 0.11). Analysis did not show any significant differences. Details are described in Table 3.

### Association between MDR1 polymorphisms and clinical characteristics of B-NHL

To evaluate the association between MDR1 polymorphisms and clinical characteristics of B-NHL, 107 B-NHL patients and 150 healthy people were enrolled. As shown in table 4, a significant association between extranodal sites and C1236T allele was observed (C vs T: P=0.01). It seemed that extranodal sites were associated with C1236T genotype (CC vs CT vs TT: P=0.03), p value was >0.05 adjusted by the Bonferroni method, however. No significant difference was found between MDR1 polymorphisms and other clinical characteristics (Table 4).

### Association between MDR1 polymorphisms and treatment-related toxicities of B-NHL

We evaluated the association between polymorphisms and treatment-related toxicities among 107 B-NHL patients. The frequencies of major hematological toxicities after chemotheraphy were compared between different alleles and genotypes of MDR1 C1236T. None of the MDR1 alleles and genotypes was associated with treatment-related toxicities (Table 5).

## Discussion

Even to the same treatment, clinical courses and responses of different B-NHL patients are variable 4,17. P-gp, encoded by MDR1 gene, plays an important role in regulating absorption, distribution, and elimination of drugs 18. The genetic variation of MDR1 gene affects the pharmacokinetics of various drugs, resulting in changes in the efficacy and side effects of the drug 18,19. Our study demonstrated that MDR1 C1236T polymorphisms were not significantly related to the risk and treatment-related toxicities of B-NHL. However, a significant association between extranodal sites and C1236T allele was observed.

Up to now, relationship between MDR1 polymorphisms and a variety of diseases have been studied 20,21. Previous study found that MDR1 Polymorphism at locus C1236T was not associated with the susceptibility of chronic lymphocytic leukemia 22, diffuse large B cell lymphoma 23 and multiple myeloma 24. Here, our study verified that MDR1 C1236T is unrelated to the occurrence of B-NHL in eastern Chinese Han population. Hui He et al. found that intermediate-risk AML patients with TTT haplotype had a lower risk of relapse than those without TTT haplotype 25. A possible reason is that the TTT haplotype is associated with a reduced P-gp expression, resulting to an increase in intracellular accumulation of chemotherapeutic agents 25. Qing Chang et al. had analyzed that it had no significant relationship between C1236T polymorphism and susceptibility to hepatocellular carcinoma in 4 cohorts 26. It seems that MDR1 polymorphisms at locus C1236T have no relationship with occurence of cancer, but closely related to recurrence of malignant tumor.

Niveditha Muralidharan et al. showed that MDR1 3435C>T gene polymorphism influences adverse events to MTX in the South Indian cohort of patients with RA 27. HYUN CHANG et al. suggested that the MDR1 genotypes at 2677 loci were associated with diarrhea in Korean advanced gastric cancer patients treated with paclitaxel-based chemotherapy 28. These relevant findings were not found in our study. We found no significant relationship between MDR1 polymorphisms and treatment-related toxicities. These studies suggested that in different population, relationship between MDR1 polymorphisms and adverse events to different drugs is different. However, detailed mechanism was not yet clear.

In conclusion, we have investigated association between MDR1 C1236T polymorphisms and B-NHL in Han population of eastern China. Extranodal sites and C1236T allele is highly correlative, but no relationship was found between MDR1 SNPs and incidence or chemotheraphy-related toxicities of B-NHL. Unfortunately, our results are lack of complete follow-up records. Thus, no survival analysis was performed. Furthermore, we may complete data of follow-up. Besides, we might pay more attention on connection between MDR1 and relevant mRNA.

## Figures and Tables

**Table 1:** Characteristics of 107 B-NHL patients in Eastern China

Characteristics	Number of patients (%)
**Sex**	
Male	56 (52.3)
Female	51 (47.7)
**Age (years)**	
≤60	56 (52.3)
>60	51 (47.7)
**Ann Arbor Staging**	
I-II	40 (37.4)
III-IV	67 (62.6)
**Group**	
A	81 (75.7)
B	26 (24.3)
**Extranodal sites**	
0-1	76 (71.0)
>1	31 (29.0)
**Performance status (ECOG)**	
0-1	85 (79.4)
≥2	22 (20.6)
**Serum LDH**	
Normal	74 (69.2)
Elevated	33 (30.8)
**IPI**	
0-2	76 (71.0)
3-5	31 (29.0)

B-NHL: B-cell non-Hodgkin lymphoma; ECOG: Eastern Cooperative Oncology Group; LDH: lactate dehydrogenase; IPI: international prognosis index.

**Table 2:** Examination of Hardy-Weinberg equilibrium of MDR1 SNPs in both B-NHL patients group and control group

Group	Observed Heterozygosity	Expected Heterozygosity	SD	*P* Value
Patients (N= 107)	0.47	0.42	0.00	0.35
Control (N= 150)	0.45	0.46	0.00	1.00

*M*DR1: Multidrug resistance gene 1; SNPs: single nucleotide polymorphisms; B-NHL: B-cell non-Hodgkin lymphoma; SD: standard deviation.

**Table 3:** Association between allele, genotype frequencies of MDR1 polymorphism and risk of B-NHL patients

Groups	Patients, n (%)	Controls, n (%)	OR	95% CI	*P* Value
**Allele**					
C	64 (29.9)	106 (35.5)	1	reference	0.20
T	150 (70.1)	194 (64.7)	1.28	0.88-1.87	
**Genotype**					
1236CC	7 (6.5)	19 (12.7)	1	reference	
1236CT	50 (46.7)	68 (45.3)	2.00	0.78-5.11	0.15
1236TT	50 (46.7)	63 (42)	2.15	0.84-5.53	0.11

*M*DR1: Multidrug resistance gene 1; B-NHL: cell non-Hodgkin lymphoma; OR: odds ratio; CI: confidence interval.

**Table 4:** Association between clinical parameters and allele, genotype frequencies of MDR1 polymorphism in patients

Characteristics	Allele			Genotype			P-Value	Pc
	C	T	P-Value	CC	CT	TT		
**Gender**								
Male	35 (31.3)	77 (68.8)	0.65	4 (7.1)	27 (48.2)	25 (44.6)	0.95	
Female	29 (28.4)	73 (71.6)		3 (5.9)	23 (45.1)	25 (49.0)		
**Age**								
≤60	37 (33.0)	75 (67.0)	0.30	3 (5.4)	31 (55.4)	22 (39.3)	0.18	
>60	27 (26.5)	75 (73.5)		4 (7.8)	19 (37.3)	28 (54.9)		
**Ann Arbor Staging**								
I-II	19 (23.8)	61 (76.3)	0.13	1 (2.5)	17 (42.5)	22 (55.0)	0.28	
III-IV	45 (33.6)	89 (66.4)		6 (9.0)	33 (49.3)	28 (41.8)		
**Group**								
A	49 (30.2)	113 (69.8)	0.85	6 (7.4)	37 (45.7)	38 (46.9)	0.79	
B	15 (28.8)	37 (71.2)		1 (3.8)	13 (50.0)	12 (46.2)		
**Extranodal sites**								
0-1	53 (34.9)	99 (65.1)	**0.01**	7 (9.2)	39 (51.3)	30 (39.5)	0.03	0.09
>1	11 (17.7)	51 (82.3)		0 (0)	11 (35.5)	20 (64.5)		
**Performance status (ECOG)**								
0-1	49 (28.8)	121 (71.2)	0.50	6 (7.1)	37 (43.5)	42 (49.4)	0.43	
≥2	15 (34.1)	29 (65.9)		1 (4.5)	13 (59.1)	8 (36.4)		
**Serum LDH**								
Normal	46 (31.1)	102 (68.9)	0.57	6 (8.1)	34 (45.9)	34 (45.9)	0.76	
Elevated	18 (27.3)	48 (72.7)		1 (3.0)	16 (48.5)	16 (48.5)		
**IPI**								
0-2	50 (32.9)	102 (67.1)	0.14	6 (7.9)	38 (50.0)	32 (42.1)	0.38	
3-5	14 (22.6)	48 (77.4)		1 (3.2)	12 (38.7)	18 (58.1)		

Data are presented as n (%) of patients. *M*DR1: Multidrug resistance gene 1; Pc: P corrected; ECOG: Eastern Cooperative Oncology Group; LDH: lactate dehydrogenase; IPI: international prognosis index. The significant p value was bolded in this table.

**Table 5:** Association between allele, genotype of MDR1 polymorphism and hematological toxicities of patients

MDR1	Leukocytopenia		Neutropenia		Anemia		Thrombocytopenia		Concurrent infection	
	0-II	III-IV	0-II	III-IV	0-II	III-IV	0-II	III-IV	0-II	III-IV
**Allele**										
C	36 (56.3)	28 (43.8)	36 (56.3)	28 (43.8)	44 (68.8)	20 (31.3)	42 (65.6)	22 (34.4)	47 (73.4)	17 (26.6)
T	86 (57.3)	64 (42.7)	70 (46.7)	80 (53.3)	102 (68.0)	48 (32.0)	90 (60.0)	60 (40.0)	103 (68.7)	47 (31.3)
P Value	0.88		0.19		0.91		0.44		0.49	
**Genotype**										
1236CC	5 (71.4)	2 (28.6)	5 (71.4)	2 (28.6)	3 (42.9)	4 (57.1)	4 (57.1)	3 (42.9)	7 (100.0)	0 (0.0)
1236CT	26 (52.0)	24 (48.0)	26 (52.0)	24 (48.0)	38 (76.0)	12 (24.0)	34 (68.0)	16 (32.0)	33 (66.0)	17 (34.0)
1236TT	30 (60.0)	20 (40.0)	22 (44.0)	28 (56.0)	32 (64.0)	18 (36.0)	28 (56.0)	22 (44.0)	35 (70.0)	15 (30.0)
P Value	0.50		0.38		0.15		0.45		0.19	

Data are presented as n (%) of patients. *M*DR1: Multidrug resistance gene 1.
